# Screening of mental health and substance users in frequent users of a general Swiss emergency department

**DOI:** 10.1186/s12873-015-0053-2

**Published:** 2015-10-09

**Authors:** Francis Vu, Jean-Bernard Daeppen, Olivier Hugli, Katia Iglesias, Stephanie Stucki, Sophie Paroz, Marina Canepa Allen, Patrick Bodenmann

**Affiliations:** Vulnerable Population Centre, Department of Ambulatory Care and Community Medicine, University of Lausanne & Lausanne University Hospital (CHUV), Rue du Bugnon 44, 1011 Lausanne, Switzerland; Alcohol Treatment Centre, Lausanne University Hospital (CHUV), Lausanne, Switzerland; Emergency Service, Lausanne University Hospital (CHUV), Lausanne, Switzerland; Institute of Social and Preventive Medicine (IUMSP) and Clinical Research Centre, Lausanne University Hospital (CHUV), Lausanne, Switzerland; Research Department, Addiction Switzerland, Lausanne, Switzerland; Department of Community Medicine and Public Health (DUMSC), Lausanne University Hospital (CHUV), Lausanne, Switzerland

**Keywords:** Emergency departments, Frequent users, Mental health disorders, Substance use disorders, Screening tools

## Abstract

**Background:**

The objectives of this study were to determine the proportions of psychiatric and substance use disorders suffered by emergency departments’ (EDs’) frequent users compared to the mainstream ED population, to evaluate how effectively these disorders were diagnosed in both groups of patients by ED physicians, and to determine if these disorders were predictive of a frequent use of ED services.

**Methods:**

This study is a cross-sectional study with concurrent and retrospective data collection. Between November 2009 and June 2010, patients’ mental health and substance use disorders were identified prospectively in face-to-face research interviews using a screening questionnaire (i.e. researcher screening). These data were compared to the data obtained from a retrospective medical chart review performed in August 2011, searching for mental health and substance use disorders diagnosed by ED physicians and recorded in the patients’ ED medical files (i.e. ED physician diagnosis). The sample consisted of 399 eligible adult patients (≥18 years old) admitted to the urban, general ED of a University Hospital. Among them, 389 patients completed the researcher screening. Two hundred and twenty frequent users defined by >4 ED visits in the previous twelve months were included and compared to 169 patients with ≤4 ED visits in the same period (control group).

**Results:**

Researcher screening showed that ED frequent users were more likely than members of the control group to have an anxiety, depressive disorder, post-traumatic stress disorder (PTSD), or suffer from alcohol, illicit drug abuse/addiction. Reviewing the ED physician diagnosis, we found that the proportions of mental health and substance use disorders diagnosed by ED physicians were low both among ED frequent users and in the control group. Using multiple logistic regression analyses to predict frequent ED use, we found that ED patients who screened positive for psychiatric disorders only and those who screened positive for both psychiatric and substance use disorders were more likely to be ED frequent users compared to ED patients with no disorder.

**Conclusions:**

This study found high proportions of screened mental health and/or substance use disorders in ED frequent users, but it showed low rates of detection of such disorders in day-to-day ED activities which can be a cause for concern. Active screening for these disorders in this population, followed by an intervention and/or a referral for treatment by a case-management team may constitute a relevant intervention for integration into a general ED setting.

## Background

In many countries (mostly in Australia, the USA, Canada, and Sweden), frequent users of emergency departments (EDs) have been a topic of interest and concern to health policy leaders and to emergency physicians, not only because of their frequent use of a costly health service but also because they often present complex medical and social needs [1–5].

Mental health and substance use issues often lead patients to seek care from ED health-care providers, hypothetically as a result of multiple factors: the 24 h availability and accessibility of ED services, the deinstitutionalization of patients, the insufficient resources or funding for community based psychiatric and substance misuse services [6, 7]. Moreover, some authors found that patients cumulating both psychiatric and substance use disorders were more likely to visit the ED compared to patients with one disorder alone (with adjusted odds ratios ranging from 2.8 to 5.6 depending on the primary disorders and the cut-off used for defining frequent ED use), although the available data did not prove the strict designation of causality [8, 9].

Compared to the ED mainstream population, ED frequent users are of particular interest because of the higher proportions of mental health and substance use disorders reported in previous studies in the latter group of patients [10–13]. Moreover, early detection of these disorders is key if appropriate inpatient and community-based interventions are to be provided. However, little is known regarding how effectively these disorders are identified in the ED setting, particularly in members of the ED frequent users population, many of whom have complex medical and social profiles leading to them seeking care for a multitude of complaints. A few studies have indeed raised concerns about missed opportunities in the ED setting to diagnose psychiatric disorders that could have been dealt with if identified [14–16].

The objectives of the present study were 1) to determine whether frequent users of an urban and general Swiss University ED had higher proportions of psychiatric and substance use disorders compared to patients who used the ED less frequently, 2) to determine whether those disorders were diagnosed in both groups of patients by ED physicians, and 3) to determine if these disorders were predictive of a frequent use of ED services.

## Methods

### Study design

This study is a cross-sectional study with concurrent and retrospective data collection at a University Hospital ED, which handles more than 45,000 ED visits annually. Between November 2009 and June 2010 (i.e. recruitment period), research assistants administered prospectively a screening questionnaire to ED patients included in the study to screen for mental health and substance use disorders and to evaluate patients’ socio-demographic and health-care use characteristics. These data were compared to data obtained from a retrospective medical chart review performed by a researcher in August 2011, searching for mental health and substance use disorders that were diagnosed by the ED physicians and recorded in the electronic medical files at the patients’ ED index visit. A pilot phase took place in October 2009, but data collected during the pilot phase were not included in this report.

### Sample and participants

The sample consisted of 399 eligible adult patients (≥18 years old) admitted to the urban, general ED of a University Hospital. Among them, 389 patients completed the researcher screening. Two hundred and twenty frequent users were compared to 169 patients who used the ED less frequently.

**Fig. 1:**
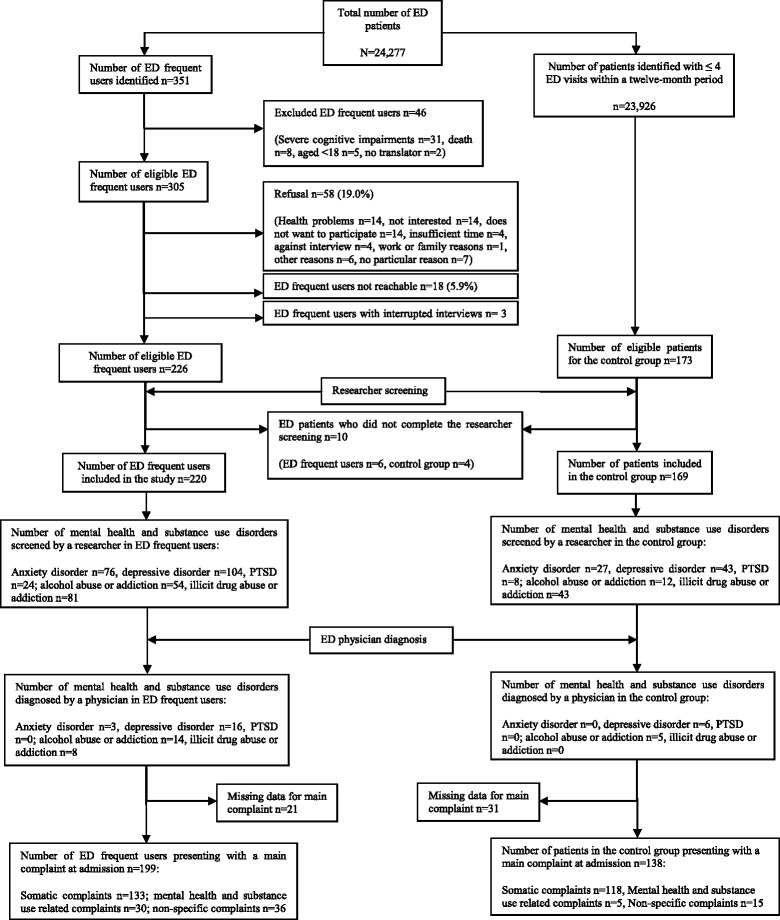
Study flow chart. ED: emergency department

Frequent users were defined as adult patients having made more than four visits to the ED in the previous 12 months, including the ED index visit (i.e. the ED visit at which the patient was recruited for the study). During the recruitment period, they were automatically identified at their ED admission by using an electronic alert system. Among the 24,277 patients who attended the general ED, 351 patients were identified as frequent users (1.5 %). Among the identified frequent users, 125 were excluded for reasons shown in detail in Fig. 1 (flow chart). Patients with one to four ED visits within a twelve-month period were randomly selected during the recruitment period using a computerized random number attribution system to comprise the control group.

Patients who attended a specialized ED (paediatric, gynaecologic, or psychiatric), who were under 18 years of age, or who had severe cognitive impairments were excluded from the study. This research was approved by the Ethics Committee of the University of Lausanne (reference number 156/09). We obtained written consent from all study participants.

### Measurements

From November 2009 to June 2010, patients’ socio-demographic, medical and health-care use characteristics were gathered prospectively in face-to-face research interviews at the patients’ index ED visit, using a screening questionnaire based on the French versions of several screening instruments which had been validated for use in a primary care setting. Mood-, panic-, and anxiety disorders were assessed using the Primary Care Evaluation of Mental Disorders (Prime-MD) [17]; post-traumatic stress disorder (PTSD) was assessed through specific parts of the Mini-International Neuropsychiatric Interview (M.I.N.I.) [18]; and alcohol and illicit drug use were identified by the Alcohol, Smoking and Substance Involvement Screening Test (ASSIST). Three research assistants (two nurses and one psychologist) were trained during the one month pilot phase to administer the questionnaire to patients.

Retrospectively, we reviewed in August 2011 the medical chart of each patient in order to extract the mental health and substance use disorders diagnosed by ED physicians and recorded in the medical file at the patient’s ED index visit. The extraction of data was performed from the hospital’s electronic file database. We also extracted the main complaints reported by patients at ED admission from their medical charts and classified them into three categories: mental health and substance use-related complaints (including alcohol intoxication, drug intoxication, and psychiatric related symptoms), somatic complaints (including ear, nose, and throat, cardiovascular, gastrointestinal, neurological, respiratory, urogenital, dermatological, and musculoskeletal symptoms), and non-specific complaints.

### Data analysis

To test the relation between qualitative data, Pearson’s chi squared test or Fisher’s exact test were used depending on the frequencies expected. For tables greater than 2*2, post hoc analyses were run and Bonferroni corrections for multiple testing were carried out. A *p*-value < 0.05 was defined as significant. All data were analyzed using STATA version 12 (StataCorp, College Station (TX), USA). To test the predictive ability of the presence of psychiatric and/or substance use comorbidity on the use of ED services, we used logistic regression analysis, controlling for age and gender.

## Results

Regarding socio-demographic characteristics, ED frequent users were younger (mean age 51.5, SD 21.5 vs mean age 56.2, SD 22.6, *p* < 0.05) and poorer (household income < CHF 3,000: 39.8 % vs 19.7 %, *p* < 0.001; unemployed or dependent on welfare: 46.5 % vs 15 %, *p* < 0.001) compared to the control group. The two groups were comparable regarding gender (female: 45.1 % vs 54.9 %, p = non-significant) and education (none, incomplete, primary: 30.2 % vs 30.1 %, p = non-significant; secondary: 51.1 % vs 47.4 %, p = non-significant; tertiary: 18.7 % vs 22.5 %, p = non-significant). More details of the socio-demographic characteristics will be presented in a separate article.

Researcher screening showed that ED frequent users had higher proportions of mental health and substance use disorders compared to patients who used the ED less frequently, as shown in detail in Tables 1, 2 and 3.

**Table 1:** Mental health and substance use disorders screened by researchers in ED frequent users compared to the control group

Screened mental health and substance use disorders	Frequent users, *n* = 220	Control group, *n* = 169	*Chi* ^*2*^ (*3*) *value*	*p*-*value*
	*n* (%)	*n* (%)		
No disorder	76 (35)	112 (67)	42.97	*p* < 0.001
Mental health disorders only	68 (31)	36 (22)		
Substance use disorders only	22 (10)	9 (6)		
Both mental health and substance use disorders	54 (25)	12 (8)

**Table 2:** Mental health disorders screened by researchers in ED frequent users compared to the control group

Screened mental health disorders		Frequent users, *n* = 220	Control group, *n* = 169	*Chi* ^*2*^ (*1*) *value*	*p*-*value*
		*n* (%)	*n* (%)		
Anxiety disorder (PRIME-MD)					
Yes		76 (34)	27 (16)	16.74	*p* < 0.001
	Non-specific anxiety disorder (F41.9)	18 (8)	9 (5)		
	Panic disorder (F41.0)	4 (2)	2 (1)		
	Panic disorder (F41.0) and non-specific anxiety disorder (F41.9)	4 (2)	0 (0)		
	Panic disorder (F41.0) and generalized anxiety disorder (F41.1)	13 (6)	3 (2)		
	Generalized anxiety disorder (F41.1)	37 (16)	13 (8)		
Depressive disorder (PRIME-MD)					
Yes		104 (47)	43 (25)	19.11	*p* < 0.001
	Other persistent mood disorder (F34.8)	20 (10)	7 (4)		
	Major depressive disorder (F32.2)	84 (37)	36 (21)		
Post-traumatic stress disorder (PTSD) (M.I.N.I.)					
Yes		24 (11)	8 (5)	4.87	*p* = 0.027

**Table 3:** Substance use disorders screened by researchers in ED frequent users compared to the control group

Screened substance use disorders		Frequent users, *n* = 220	Control group, *n* = 169	*Chi* ^*2*^ (*1*) *value*	*p*-*value*
		*n* (%)	*n* (%)		
Alcohol abuse or addiction (ASSIST)					
Yes		54 (24)	12 (7)	21.12	*p* < 0.001
	Alcohol abuse (F10.1)	35 (16)	11 (6)		
	Alcohol addiction (F10.2)	19 (8)	1 (1)		
Illicit drug abuse or addiction (ASSIST)					
Yes		81 (36)	43 (25)	19.11	*p* < 0.001
	Drug abuse (F1x.1)	32 (14)	29 (17)		
	Drug addiction (F1x.2)	49 (22)	14 (8)		

**Fig. 2:**
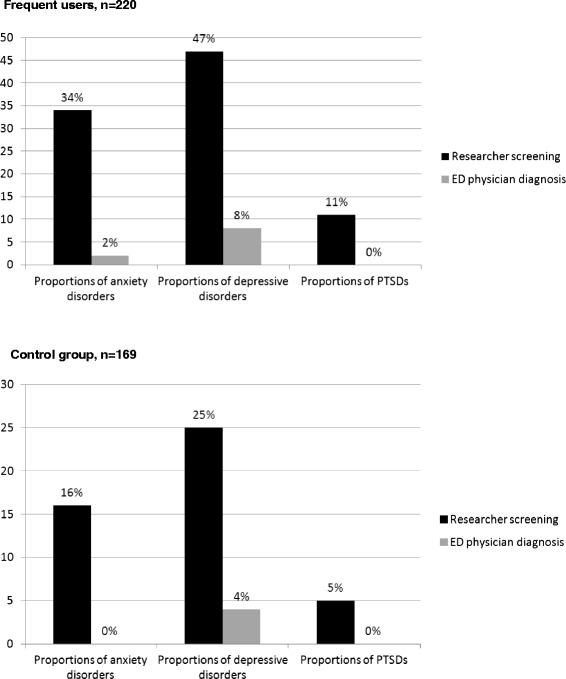
Mental health disorders in ED frequent users and in the control group: researcher screening vs ED physician diagnosis

**Fig. 3:**
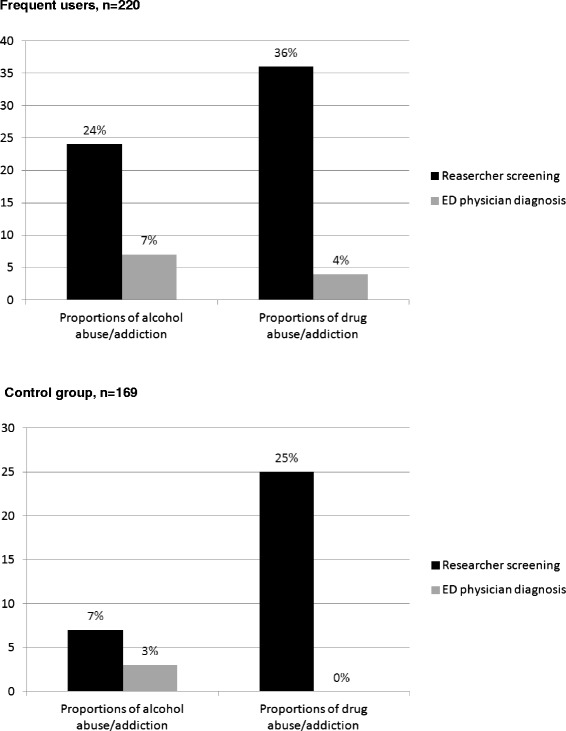
Substance use disorders in ED frequent users and in the control group: researcher screening vs ED physician diagnosis

Reviewing the ED physician diagnosis, we found that the proportions of mental health and substance use disorders diagnosed by ED physicians at the patients’ ED index visit were low in both ED frequent users and the control group, as shown in detail in Figs 2 and 3. There was no significant difference in the detection rate for mental health and substance use disorders between ED frequent users and the control group (Fisher’s Exact Test: anxiety, *p* = 0.567; depression, *p* = 1.000; PTSD, *p* = 1.000; alcohol, *p* = 0.517; and drugs, *p* = 0.053). We also found that ED frequent users were more likely to make an ED visit for mental health and substance use-related main complaints compared to the control group (Chi^2^ (1) = 12.12, *p* < 0.001, missing data for 27 ED frequent users and 35 patients in the control group). No difference was found regarding somatic and non-specific main complaints.

Using multiple logistic regression analyses to predict frequent ED use, we found that ED patients who screened positive for psychiatric disorders only were more likely to be ED frequent users compared to those with no disorder (adjusted odds ratios [ORs] of 2.6 [95 % CI 1.5 to 4.5], *p* = 0.001). Moreover, ED patients who screened positive for both psychiatric and substance use disorders were at higher risk of being ED frequent users compared to those with no disorder (ORs of 4.9 [95 % CI 2.6 to 9.1], *p* < 0.001). No difference was found when comparing ED patients who screened positive for substance use disorders only to those with no disorder. ORs were controlled for age and gender.

## Discussion

In this study, researcher screening showed that ED frequent users had higher proportions of mental health and substance use disorders compared to patients who used the ED less frequently. Reviewing the ED physician diagnosis, we found that the proportions of these disorders diagnosed by ED physicians were low both among ED frequent users and in the control group. The performed statistical analysis showed that ED patients who screened positive for psychiatric disorders only and those who screened positive for both psychiatric and substance use disorders were more likely to be ED frequent users compared to ED patients with no disorder.

Regarding the researcher screening, we found higher proportions of mental health and substance use disorders in ED frequent users compared to the ED mainstream population (no disorder: 35 % vs 67 %, mental health disorders only: 31 % vs 22 %, substance use disorders only: 10 % vs 6 %, both mental health and substance use disorders: 25 % vs 8 %; Chi^2^ (3) = 42.97, p < 0.001). Previous studies have reported similar observations in the UK [13], in the USA [12] and in Canada [11]. In Switzerland, a retrospective chart review case–control study mentioned that mental health (9.4 % vs 2.0 %, *p* < 0.001) and substance abuse (12.3 % vs 6.5 %, *p* = 0.02) diagnoses were made more often in the case of ED frequent users compared to less frequent users [10]. While the proportions of disorders reported for each group of patients differ across these studies, some factors intrinsic to the studies’ design may explain these differences.

First, higher proportions of psychiatric and substance use disorders were found in studies [13], including the present study, in which screening instruments were administered to patients by physicians or research assistants. Retrospectively designed studies reported lower proportions of mental health and substance use disorders [10–12]. This suggests that an active search by health-care providers using validated screening instruments may facilitate the identification of such disorders [19].

Second, the cut-off used in defining frequent ED use may contribute to the differences in the proportions of mental health and substance use disorders found in ED frequent users compared to patients who visited the ED less frequently. As previously mentioned, mental health and substance use problems are associated with a frequent use of ED services [6, 7]. Conversely, it may be that the higher the number of ED visits, the higher the probability a patient may suffer from a psychiatric and/or substance use disorder. Studies with a cut-off set at ≥ 7 ED visits within a twelve-month period [11, 13] showed higher proportions of mental health and substance use disorders in ED frequent users compared to studies with a cut-off set at ≥ 4 or 5 ED visits within a twelve-month period [10, 12]. While there is no clear consensus on defining frequent ED use, with thresholds used ranging from two to more than 12 ED visits within a twelve-month period [5], we chose > 4 ED visits within the previous 12 months as a cut-off as it may correspond to non-random events according to Locker et al. [20].

Regarding the proportions of mental health and substance use disorders diagnosed by ED physicians, they were low both in the group of ED frequent users and in the group of patients who visited the ED less frequently. In our opinion, two main factors should be considered as they may minimize or prevent the recognition of mental health and substance use disorders: the appropriateness and the feasibility of a screening in the ED setting.

The appropriateness of generalized screening for mental health and substance use disorders among ED patients is a question that may remain controversial and unresolved, as some health-care providers would consider the ED as an environment primarily dedicated to addressing acute and short-term health problems. In that perspective, screening for these disorders may be viewed as a time- and energy-consuming process that would not fit into the mission of a chronically overcrowded and under pressure health service [21]. This issue is compounded by the numerous other screening interventions that have been advocated to EDs during the last three decades: HIV, intimate partner violence, risk of fall injuries in the elderly, tobacco use, etc. [22]. On the other hand, other health-care providers and public health professionals may see a general ED as a setting in which a holistic and long-term health-care plan can be initiated. Some authors are indeed concerned about opportunities for diagnosing psychiatric disorders in the ED setting [15, 16]. In this perspective, an ED visit may constitute an opportunity for the early recognition of such disorders, preventing further complications by enabling the initiation of an appropriate treatment. Generalized screening for mental health and substance use disorders in an ED setting should also be viewed in the light of its feasibility. As emphasized by some authors [14], new screening instruments may need to be developed and validated if they are to be used in day-to-day ED activities. Adapted and trained resources would then be needed on the ED staff to ensure that patients screened positively would be adequately treated [14], raising concerns regarding additional costs for ED services.

Regarding whether mental health and substance use disorders may be predictive of a frequent use of ED services, our study showed that patients who screened positive for psychiatric disorders only and those who screened positive for both psychiatric and substance use disorders were at higher risk of visiting an ED frequently (>4 visits within a twelve-month period) compared to patients with no disorder, regardless of their age or gender. These results seem to concur with observations found in previous studies, as mental health and substance users may seek health-care in ED services, hypothetically related to their 24 h availability and accessibility, rather than using community based services whose access may be more limited or more difficult [6, 7]. This seems to be especially true for uninsured patients [6]. The frequent use of EDs may be also the consequence of behavioural or social characteristics in this population of patients that warrant future research to determine the precise underlying mechanisms leading them to use EDs rather than other services [6, 7]. Based on our observations, we could find no explanation on why substance use disorders only were not predictive of a frequent ED use.

Overall the evidence strongly suggests that screening the ED frequent user population for mental health and substance use disorders may constitute a relevant intervention due to the significantly higher proportions of these disorders in that group of patients compared to the mainstream ED population. Furthermore, such an intervention may help identify ED patients with silent disorders that could be dealt with if diagnosed [14–16]. In 2004, 5 % of the general Swiss population had a depression–or bipolar disorder, 10 % an anxiety disorder, 0.5 % a psychotic disorder, and 2 % an addiction (illicit drug or alcohol) disorder [23]. Psychiatric and substance use problems generated an estimated overall cost of more than 11 billion Swiss francs per year mainly made up of indirect costs such as those related to absenteeism or early retirement [24].

However, as mentioned earlier, EDs may not have the resources needed to implement an active screening of mental health and substance use disorders in ED frequent users, nor to provide the following steps if disorders were to be identified such as referring patients to community based services, insuring their access to outpatient treatments, etc. In this perspective, the intervention of a case management team could help front-line health-care providers in improving the clinical management of this group of patients by performing such an active screening. US case management programs have shown significant benefits for the management of ED frequent users by improving their clinical and social outcomes as well as reducing their ED use and, hence, ED costs [25] and our local University Hospital implemented such a case management program in 2009, as fitted the reality on the ground [10]. The case management team in our program consisted of nurses who worked under the supervision of general practitioners and who acquired clinical skills (such as motivational interviewing skills, transcultural communication skills, etc.) that helped ED health-care providers respond to the patients’ medical and social needs. Interventions made by our case managers were, for instance, to refer patients to the appropriate primary care services, to improve communication and collaboration within their healthcare and social network, to provide them general health counselling. To assess the clinical and economical impact of such an intervention, we started in April 2012 a randomized controlled trial in our local hospital with results being processed at the moment.

A future development of screening instruments for mental health and substance use disorders adapted to the ED setting may be of particular interest for our case management team to improve the detection of such disorders in the ED frequent users population.

### Limitations

The observations and results in the current study are limited to a single urban, general ED of a teaching hospital, and therefore are not representative of other rural or urban Swiss ED sites or of foreign EDs.

To collect the ED physicians’ mental health and substance misuse diagnoses, we chose to review the patients’ electronic medical files because it was an easier and less time-consuming method rather than asking the ED physicians to complete a survey or to administer themselves the screening questionnaire to the patients. This method of collection allowed the reflection of the true diagnostic process used by ED physicians in their day-to-day activities. Another advantage is the avoidance of recall biases that may be observed when surveys are performed [26]. A disadvantage of the collection method we used is that mental health and substance use disorders may have been previously known of, suspected, or identified by ED physicians but not recorded in the medical files at the ED index visit. Therefore, clinically diagnosed mental health and substance use disorders may have been underestimated in our results.

The screening instruments used have their intrinsic limitations (e.g. they have been validated only for the primary care setting) and may need to be adjusted and validated for use in an ED setting. Moreover, the screening instruments we used allowed only for the identification of a limited selection of psychiatric disorders and were not inclusive of other mental illnesses (such as psychotic related disorders). Consequently, the overall burden of psychiatric disorders was probably underestimated in our study.

## Conclusions

In this study, frequent users of an urban, general Swiss ED had higher proportions of mental health and substance use disorders compared to the mainstream ED population, and the proportions of these disorders diagnosed by the ED staff were low in both groups of patients. A predictive factor of the frequent use of ED services by patients was the presence of psychiatric disorders only or the presence of both psychiatric and substance use disorders. Although studies have, for over a decade, been reporting high proportions of such disorders in ED frequent users, their identification in a general ED setting remains a cause for concern. Considerations regarding the appropriateness and the feasibility of screening for mental health and substance use disorders in a general ED warrant future research. Nonetheless, active screening for these disorders among ED frequent users by a case-management team could improve the response to the evolving needs of the ED population by helping front-line health-care providers improve the clinical management of ED frequent users.

## Abbreviations

ASSIST: ALCOHOL, SMOKING AND SUBSTANCE INVOLVEMENT SCREENING TEST
ED: Emergency department
M.I.N.I.: Mini-International Neuropsychiatric Interview
Prime-MD: Primary Care Evaluation of Mental Disorders
PTSD: Post-traumatic stress disorder
